# Intra-abdominal infection with *Campylobacter curvus*: case report and review of the literature

**DOI:** 10.1099/acmi.0.000227

**Published:** 2021-04-21

**Authors:** Patrick C. K. Tam, Lex E. X. Leong, Maria Theodossi, David L. Gordon

**Affiliations:** ^1^​ Departments of Microbiology and Infectious Diseases, Flinders Medical Centre, Adelaide, South Australia, Australia; ^2^​ College of Medicine and Public Health, Flinders University, Adelaide, South Australia, Australia; ^3^​ SA Pathology, Adelaide, South Australia, Australia; ^4^​ Clinical & Health Sciences, University of South Australia, Adelaide, South Australia, Australia; ^5^​ Microbiome Research, South Australian Health and Medical Research Institute, Adelaide, South Australia, Australia

**Keywords:** *Campylobacter curvus*, extra-oral infection, whole genome sequencing, treatment

## Abstract

**Background:**

*
Campylobacter curvus* is a Gram-negative bacteria associated with periodontal disease in humans. Cases of extra-oral manifestations of infection are rare with only six reported cases of extra-oral infection including this report that have been identified in the current literature. Molecular methods are generally used to identify *C. curvus* while optimal antibiotic choice and duration to treat extra-oral infections for this pathogen is unknown.

**Case presentation:**

A 63-year-old male with a background history of alcoholic pancreatitis presented with fever and malaise who was found to have radiological intra-abdominal collections. Drainage of these collections identified *C. curvus* via matrix-assisted laser desorption/ionisation time of flight (MALDI-TOF) mass spectrometry with high probability and identification further confirmed by whole-genome sequencing. Antibiotic susceptibility testing to erythromycin and ciprofloxacin of *C. curvus* was performed using E-test diffusion methods along with investigation for the presence of resistance genes. The patient was treated with intravenous piperacillin-tazobactam followed by ciprofloxacin for 4 weeks total with good clinical recovery.

**Conclusions:**

Extra-oral manifestations with the pathogen *C. curvus* are rare with few cases described in the literature. There is minimal data on susceptibility patterns, optimal antibiotic treatment and duration. Treatment of extraintestinal *C. curvus* infections in humans should encompass both adequate source control and antibiotic therapy.

## Background

The *Campylobacter* genus comprises a large group of over 30 bacterial species with its more familiar members *Campylobacter jejuni* and *Campylobacter coli* implicated mainly in gastrointestinal disease [1]. Extra-intestinal infection with *Campylobacter* spp. including bacteremia, urinary tract infections and extraluminal collections are also known to occur with lesser-known members in the genus such as *Campylobacter curvus* emerging as pathogens causing infections in humans [2]. Originally identified as *Wolinella curva* in 1984 from human oral samples, *C. curvus* is a motile curved Gram-negative bacillus [3–5]. Following immunotypic and genetic analyses, *W. curva* was revised to *C. curvus* given homology to other *Campylobacter* species [6]. Notable for its association in periodontal disease and sporadic infectious diarrhoea, extra-oral infections with *C. curvus* are rare [7]. A review of the literature demonstrates only five case reports of extra-oral infection to date [8–11]. We describe here a case of *C. curvus* intra-abdominal infection including the methodology for identification and management as well as a comparison to the cases identified in the literature for management of extra-oral infections with this species.

## Case report

A 63-year-old male presented with 4 weeks of malaise and fever in the setting of a prior pancreatic pseudocyst co-infection with *Enterococcus faecalis, Klebsiella pneumoniae* and mixed coagulase negative staphylococci, on a background of alcoholic pancreatitis. This infected pseudocyst had been drained followed by 6 weeks of intravenous piperacillin-tazobactam and a further 2 weeks of oral ciprofloxacin with amoxicillin-clavulanate. Treatment was completed 5 months prior with complete clinical recovery, marked radiological reduction in the collections and resolution of inflammatory markers. His medical history was also notable for hypertension and alcoholic cardiomyopathy.

**Fig. 1.**
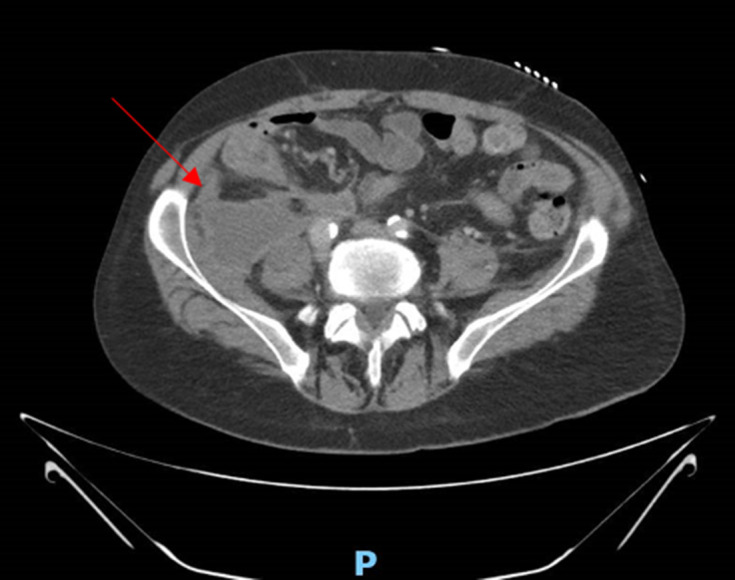
Axial view of a computed tomography scan of the abdomen at presentation demonstrating one of the large intra-abdominal collections measuring 129×58 mm.

Examination at representation to hospital demonstrated right basal crackles in the lungs and a non-tender abdomen. Investigations demonstrated an elevated white cell count of 16.33×10^9^ per litre with a predominant neutrophilia and a C-reactive protein of 156 mg l^−1^ (normal limits: 0–8 mg l^−1^). Other abnormalities included sodium, potassium and creatinine levels of 123 mmol l^−1^, 5.3 mmol l^−1^, and 201 μmol l^−1^ respectively. Liver function tests remained normal with a normal lipase level of 36 U l^−1^. A computed tomography (CT) scan of his abdomen showed interval progression of pre-existing retroperitoneal and intraperitoneal collections with the largest collection measuring 129×58 mm (Fig. 1).

Ceftriaxone and metronidazole were commenced empirically, and CT-guided drains were inserted into the largest two collections. Sixty millilitres of purulent specimen was aspirated. Although there were no immediate complications, the patient later developed hypotension secondary to the underlying cardiomyopathy and transferred to the intensive care unit. Antimicrobial therapy was changed to piperacillin-tazobactam. Following haemodynamic stabilisation, he returned to the surgical ward 48 h later. A repeat CT scan showed near resolution of the two main collections.

**Fig. 2.**
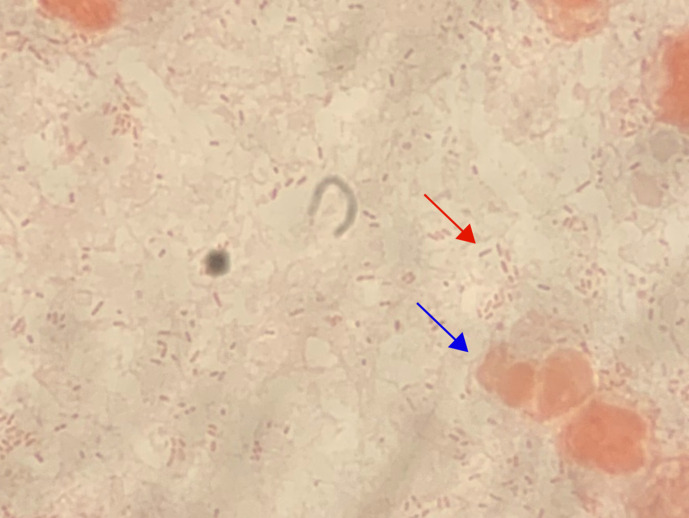
Gram-stain demonstrating small Gram-negative bacilli (red arrow) in the presence of polymorphonuclear cells (blue arrow) on high power field microscopy.

**Fig. 3.**
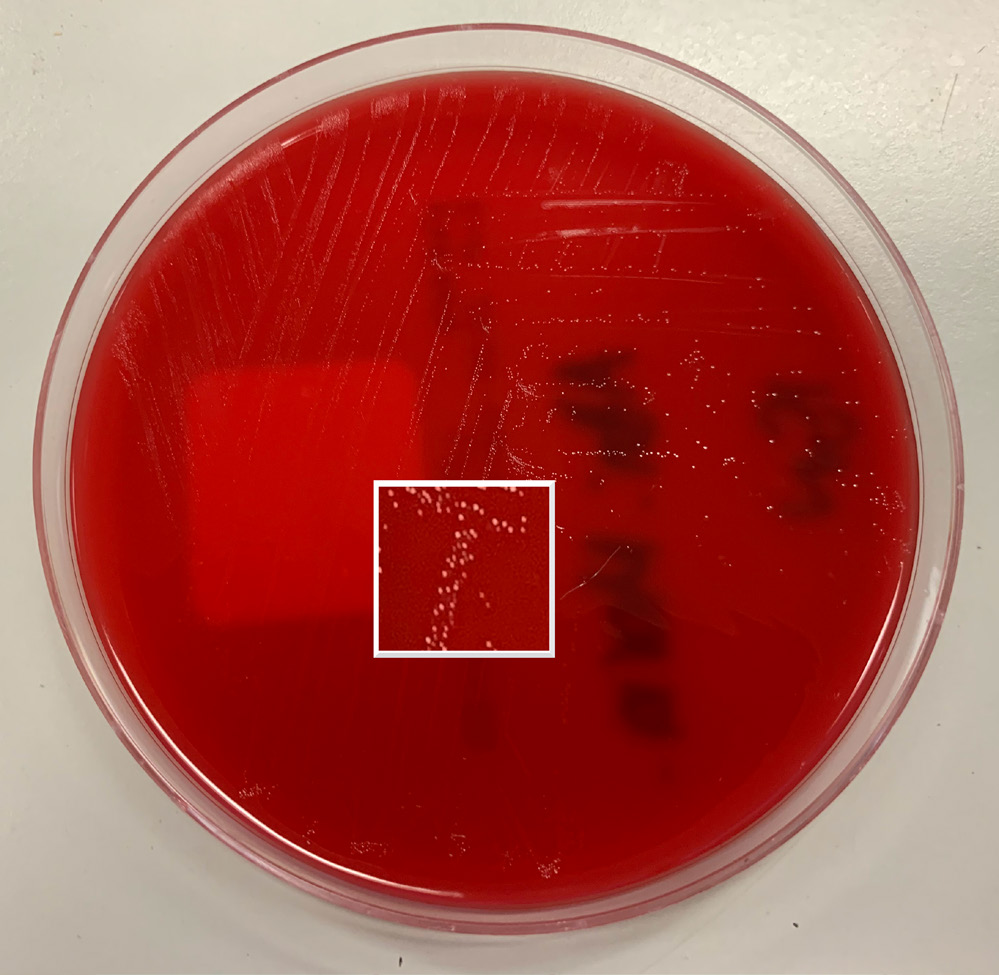
Growth of tiny white colonies after 48 h on horse blood agar under anaerobic conditions at 37 degrees Celsius with section magnified.

Gram-stain of aspirated material demonstrated numerous polymorphic leukocytes and Gram-negative bacilli under high powered field microscopy (Fig. 2). After 2 days of incubation, tiny white non-haemolytic colonies were cultured on horse blood agar under anaerobic conditions at 37 degrees Celsius (Fig. 3). The organism was oxidase-positive, catalase-negative, H_2_S non-producing, urease-negative, and motile. Using matrix-assisted laser desorption/ionisation time of flight (MALDI-TOF) mass spectrometry (Bruker; Preston, Victoria), the isolate was identified as *C. curvus* with a score of 2.143.

Genomic DNA was extracted from the cultured colonies and amplicon libraries prepared using Nextera XT Library Preparation kit. Sequencing was performed on a NextSeq 500 platform with a NextSeq Mid-Output (2×150 bp) kit (Illumina Inc., CA, USA). Raw FASTQ files were used to construct draft genomes using shovill (v1.0.9), and genome annotation was performed using prokka (v1.14.6) [12]. Pangenome analysis was performed using roary (v3.13.0) with 90 % minimum percentage identity for BLASTP. Whole-genome sequencing supported speciation of *C. curvus *. Abricate (v1.0.1) was used to detect antibiotic resistance genes from the genomic sequence, however, no antimicrobial resistance genes could be detected from the draft genome.

Antibiotic testing using E-test diffusion methods showed a ciprofloxacin and erythromycin minimum inhibitory concentration (MIC) of 0.19 ug ml^−1^ and 16 ug ml^−1^ respectively. The patient completed 10 days of intravenous piperacillin-tazobactam followed by oral ciprofloxacin for a further 3 weeks. At the time of antibiotic cessation, the patient had near radiological resolution of the drained collections and normalisation of his inflammatory markers. He remained clinically stable 2 months post-antibiotic cessation following hospital discharge.

## Discussion

*Campylobacter* spp. are a group of bacteria with its most familiar members *C. jejuni* and *C. coli* known to cause primarily gastrointestinal infection in humans [13]. Lesser-known species in the genus such as *C. curvus* are emerging as potential pathogens causing human disease. *C. curvus* is a non-fermenting, oxidase positive, microaerophilic Gram-negative bacillus that produces pinpoint colonies on blood agar when cultured under microaerophilic conditions [7]. Further biochemical characteristics of *C. curvus* are shown in Table 1. Isolation of *C. curvus* in human samples outside the oral cavity is rare and its relationship to human pathology is poorly understood [3]. Found as commensal bacteria in the human mouth, notably periodontal pockets of diseased gums, a causal role for these organisms in periodontal disease has not been clearly established [14].

**Table 1.** Summary of biochemical properties of *C. curvus
*

Catalase	Urease	Nitrite reduction	H_2_ requirement for growth	H_2_S production	Indole acetate hydrolysis	Hippurate	Oxidase	Motility
+/-	−	+	+	+	+	−	+	+

References: [26–28].

Humans are known hosts to *C. curvus* with rates of carriage presumed low. One study of human faecal specimens investigating presumed gastroenteritis identified *C. curvus* in only one of 320 samples [15]. In a similar microbiome study, *C. curvus* DNA from intestinal biopsies was found in only 1.4 and 4.6 % of patients with ulcerative colitis and healthy controls respectively [16]. *C. curvus* as a cause of infectious diarrhoea is rare with *C. curvus* isolated in only 0.0004–0.05 % of children with infectious gastroenteritis [3, 17]. This was similar to another study of infectious diarrhoea whereby *C. curvus* was isolated in only 15 patients over a 4 year period [7].

Extra-oral infection with *C. curvus* is rare. A search of the literature using the PubMed and EMBASE library databases for articles on *C. curvus* that have been published to date was conducted. Search terms included ‘* Campylobacter curvus *’ OR ‘* Wolinella curva *’. Twenty-three and sixty-three results based on these terms were found in the PubMed and Embase databases respectively with only five case reports of extra-oral infection, comprising two cases of liver abscesses, two cases of pulmonary infection and a case of chorioamnionitis (Table 2) [8–11].

**Table 2.** Summary of clinical characteristics, risk factors, diagnostics and management of patients with extra-oral *Campylobacter curvus* infections

Case	Patient’s sex and age	Diagnosis	Risk factors	Identification technique	Time to isolation	Associated organisms	Intervention	Antibiotic therapy (duration)	Outcome	Ref
**1**	52M	Liver abscess	Diverticulitis	16 s RNA gene sequencing	3 days	Monomicrobial	Radiological drainage	ceftriaxone+metronidazole (unknown)	Survived	[8]
**2**	68F	Liver abscess	Ovarian cancer	16 s RNA gene sequencing	unknown	*Alpha-hemolytic Streptococcus* spp	Radiological drainage	piperacillin-tazobactam (6 weeks)	Survived	[9]
**3**	59M	Lung abscess	Lung cancer	16 s RNA gene sequencing	unknown	* Streptococcus constellatus *	Lung resection	Unknown	Survived	[9]
**4**	65F	Empyema	Bronchiectasis	16 s RNA gene sequencing	2 days	* Peptostreptococcus *	Chest drain	ampicillin/sulbactam+clindamycin (5 weeks)	Survived	[10]
**5**	29F	Chorioamnionitis	Pregnancy	Molecular diagnostics (not specified)	unknown	*H parainfluenza*	Delivery	IV amoxicillin, gentamicin and metronidazole (2 days)	Both survived	[11]
**6**	64M	Intra-abdominal collection	Chronic pancreatitis	MALDI-TOF, whole genome sequencing	2 days	Monomicrobial	Radiological drainage	piperacillin-tazobactam (10 days) → PO ciprofloxacin (3 weeks)	Survived	This case

In four cases, *C. curvus* was isolated on bacterial media when cultured under anaerobic conditions. All five cases prior to this report required molecular detection methods for identification of *C. curvus* with 16S rRNA sequencing the main method used. Three cases with infected collections were polymicrobial and in the case of *C. curvus-*associated chorioamnionitis, *Haemophilus parainfluenza* was also detected via molecular methods. In contrast to predominantly monomicrobial infections with *C. foetus*, *C. curvus* appears to exhibit co-habitation with other bacteria leading to polymicrobial infection similar to *C. showae* [13, 18]. Whilst *C. curvus* was the only pathogen isolated in this patient’s collection, this may be attributable to pre-treatment with antibiotics or selective pressures from previous therapy.

**Fig. 4.**
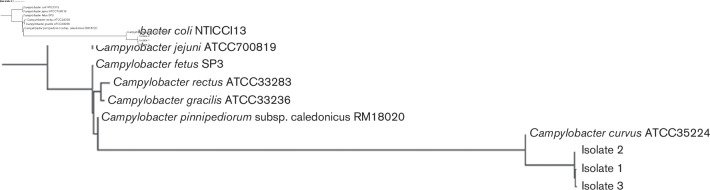
Phylogenetic tree of isolate recovered from the reported case with other known *Campylobacter* species. The maximum likelihood tree was constructed using alignment of gene-to-gene comparison.

Whilst prior studies relied on molecular techniques to identify *C. curvus *, the isolate from this study was identified using MALDI-TOF MS with a high probability score. MALDI-TOF MS correctly identifies up to 91 and 83 % of all non-*jejuni/coli Campylobacter* strains and *C. curvus* isolates respectively [19]. The isolate demonstrated identification of *C. curvus* with a score of 2.143 suggesting a high probability match. Whole-genome sequencing was used to confirm speciation and based on the genomic context from pan-genomes against other *Campylobacter* spp., the isolate appeared most related to *C. curvus* (Fig. 4).

Susceptibility testing and antibiotic MIC reports for *C. curvus* strains are limited due to the rarity of the infection. Reported MICs in the literature are variable but include: bacitracin >128 µg ml^−1^, ceftriaxone >32 µg ml^−1^, cefuroxime >256 µg ml^−1^, chloramphenicol 2–4 µg ml^−1^, ciprofloxacin 0.047 µg ml^−1^, clindamycin 0.5–1 µg ml^−1^, erythromycin 2 µg ml^−1^, gentamicin 2 µg ml^−1^, metronidazole 0.38–2 µg ml^−1^, and penicillin 32 µg ml^−1^ [4, 8]. Susceptibility to β-lactams in *Campylobacter* spp are variable with minimal data in particular to piperacillin-tazobactam [20]. One study examining C. *coli */C. *jejuni* found low resistance to amoxicillin but high resistance to piperacillin thought secondary to poor penetration due to the bulky side chain in the latter [21].

E-test diffusion testing of the isolate demonstrated an MIC of 0.19 µg ml^−1^ for ciprofloxacin and 16 µg ml^−1^ for erythromycin. Although interpretation for these MIC values is not available for non-jejuni/coli *Campylobacter spp* due to limited data on *C. curvus *, we interpreted the isolate to be ciprofloxacin susceptible and erythromycin resistant by extrapolating EUCAST breakpoints for *C. jejuni/C. coli* species [22]. No gene could be detected in the draft genome that would confer antimicrobial resistance. Nevertheless, the draft genome harboured several sets of virulence genes encoding components of the bacterial flagellar system (*fliG, fliN, fliM, fliS, fliW, fliE, fliP, fliQ, fliR, flhA, flhB, flgB, flgC,* and *flgG*), flagellin A (*flaA*), and a secreted flagellin C (*flaC*). Notably, resistance via upregulation of motility and down regulation of metabolism following treatment with erythromycin has been found in C. *jejuni* [23].

Limited data exists for *C. curvus* susceptibility to erythromycin but this isolate appeared consistent to other non-*jejuni/coli* species that show erythromycin resistance. This is potentially through mutations in the 23S RNA or via efflux pumps such as *cmeABC* [20, 24]. The gene cluster *cmeABC*, which confers intrinsic low level erythromycin resistance in *C. coli/jejuni*, was not detected in the isolate in this study but detection may have been hampered by the genetic distance between *C. curvus* and *C. jejuni/coli* or limited characterisation studies for this species of campylobacter.

The patient in this case report continued antibiotic therapy with ciprofloxacin following broad-spectrum antibiotics. Although C. jejuni*/coli* species readily acquire resistance to fluoroquinolones limiting its use, whether this applies to *C. curvus* is unclear [24]. The optimal duration of treatment following source control is uncertain, ranging between 2 days to 6 weeks. Given the propensity for *C. curvus* to form biofilms and its persistence in inhospitable environments, a prolonged treatment course may be prudent to ensure clearance of infection. The patient was treated with 3 weeks of ciprofloxacin following drainage [25].

## Conclusion

Extraoral manifestations of *C. curvus* are rare with infections predominantly due to polymicrobial collections. Identification of *C. curvus* is classically reliant on molecular techniques due to its fastidious nature but the MALDI-TOF MS may be adequate in identifying most *C. curvus* isolates. Management of extraoral infections require good surgical debridement or removal of the infection with a prolonged treatment course of antibiotics.
